# The transversus abdominis plane block in conjunction with intrathecal morphine use after cesarean section in women with severe pre-eclampsia: a randomized controlled trial

**DOI:** 10.1186/s12871-023-02061-9

**Published:** 2023-03-30

**Authors:** Zhi-rong Yan, Li-juan Chen, Su-jing Zhang, Long-xin Zhang, Huan Lu, Li Zhang, Ming Liu, Min Zhou, Li-hua Lin

**Affiliations:** 1 Department of Anesthesiology, College of Clinical Medicine for Obstetrics & Gynecology and Pediatrics, Fujian Key Laboratory of Women and Children’s Critical Diseases Research, Fujian Maternity and Child Health Hospital, Fujian Medical University, Fuzhou, Fujian Province 350001 China; 2 Department of Child healthcare center, College of Clinical Medicine for Obstetrics & Gynecology and Pediatrics, Fujian Key Laboratory of Women and Children’s Critical Diseases Research, Fujian Maternity and Child Health Hospital, Fujian Medical University, Fuzhou, Fujian Province 350001 China; 3 Department of Healthcare, College of Clinical Medicine for Obstetrics & Gynecology and Pediatrics, Fujian Key Laboratory of Women and Children’s Critical Diseases Research, Fujian Maternity and Child Health Hospital, Fujian Medical University, 18 Dao shan Road, Fuzhou, Fujian 350001 China

**Keywords:** Severe pre-eclampsia, Cesarean section, Intrathecal morphine, Transversus abdominis plane, Postoperative analgesia

## Abstract

**Background:**

The transversus abdominis plane (TAP) block in conjunction with intrathecal morphine has been demonstrated to provide more superior postcesarean analgesia to intrathecal morphine alone. However, the analgesia efficacy of their conjunction has not been demonstrated in patients with severe pre-eclampsia. The study aimed to compare the postcesarean analgesia of TAP block in conjunction with intrathecal morphine versus intrathecal morphine alone in women with severe pre-eclampsia.

**Methods:**

Pregnant women with severe pre-eclampsia undergoing planned cesarean section were randomly allocated into 2 groups to receive TAP block with 20 ml of 0.35% Ropivacaine (TAP group) or with the same volume of 0.9% saline (Sham group) after undergoing elective cesarean section under spinal anaesthesia with 15 mg of 0.5% Ropivacaine plus 0.1 mg of morphine. The outcomes for this analysis include the visual analog scale (VAS) pain score at rest and with movement at 4,8,12,24 h after TAP block was performed, times of use of intravenous patient-controlled analgesia (PCA) within 12 h after anesthesia, the occurrence of maternal side effects, maternal satisfaction, and Apgar score at 1 and 5 min of newborns.

**Results:**

119 subjects receive TAP block with 0.35% Ropivacaine (n = 59)or 0.9% saline (n = 60). At 4,8, 12 h after TAP block, the TAP group reported lower VAS score at rest [at 4 h: 1(0,1) vs. 1(1,2), *P < 0.001*; at 8 h:1(1,1) vs. 1(1.5,2),*P < 0.001*; at 12 h:1(1,2) vs. 2(1,2),*P = 0.001*] and higher satisfaction [53(89.9%) vs.45(75.0%), *P < 0.05*]. There were no differences between groups in VAS score at 24 h at rest and at all time points above with movement, times of use of PCA within 12 h after anesthesia, maternal side-effect, and Apgar score at 1 and 5 min of newborns.

**Conclusions:**

In conclusion, The TAP block performed in conjunction with intrathecal morphine may not reduce opioid consumption, but it could reduce VAS scores at rest in the first 12 h after cesarean section in women with severe pre-eclampsia, and improve maternal satisfaction, which is worthy of clinical promotion.

**Trial registration:**

Registered at Chinese Clinical Trial Registry(http://www.chictr.org.cn) on 13/12/2021: ChiCTR2100054293.

## Background

Severe pre-eclampsia is a complicated obstetrical disease, which can threaten the health of the maternal and fetus and is usually terminated by cesarean Sects. [1, 2]. The analgesia management including postcesarean analgesia of severe pre-eclampsia is critical. Poor pain control after cesarean delivery can cause a series of pathophysiological changes to the mother, even causing cerebrovascular accidents and eclampsia[1]. Women with severe pre-eclampsia are in a state of anxiety long-term before surgery, have a low pain threshold, and require more complete postcesarean analgesia[3]. Intrathecal morphine is considered the gold standard for postcesarean analgesia, which can reduce the amount of postoperative intravenous opioids and the side effects caused by them[4, 5]. However, with the development of anesthesia, multimodal analgesia is widely promoted[6–8].

Transversus abdominis plane (TAP) block is an easy operated nerve block to control pain after abdominal surgery, particularly among patients undergoing cesarean Sects. [9–11].Moreover, the efficacy of the postcesarean analgesia of their combined use in women with severe pre-eclampsia has not been explicitly studied. In addition, we wondered whether the combined use could reduce the incidence of attendant opioid-related side effects such as chills, nausea, respiratory depression, and itching. We hypothesized that the TAP block in conjunction with intrathecal morphine would provide better superior postcesarean analgesia in women with severe pre-eclampsia when compared with intrathecal morphine alone. We conducted a prospective randomized controlled trial to compare the analgesic effects of intrathecal morphine combined with TAP block after cesarean delivery in severe pre-eclampsia. The primary outcome of our study was the difference in visual analog scale (VAS) pain score at rest and with movement within 24 h after TAP block was performed and the times of use of intravenous patient-controlled analgesia (PCA) infusion device within 12 h after anesthesia. The secondary outcome was maternal analgesia satisfaction, occurrence of side effects within 24 h after anesthesia, maternal satisfaction, maternal side-effect, and Apgar score at 1 and 5 min of newborns.

## Methods

This prospective randomized controlled trial was approved by the Ethics Committee of Fujian Maternity and Child Health Hospital(NO.2021KLRD09042) and registered the study at Chinese Clinical Trial Registry on 13/12/ 2021 (Registration number: ChiCTR2100054293). Informed consent was obtained from all patients before enrollment. This manuscript adheres to the applicable CONSORT guidelines. The authors assert that all procedures contributing to this work comply with the ethical standards of the relevant national and institutional committees on human experimentation and with the Helsinki Declaration of 1975, as revised in 2008. During Jan 2022 and Jun 2022, we recruited 120 patients with severe pre-eclampsia who scheduled for elective cesarean section and planned spinal anaesthesia. The eligibility criteria include following: singleton pregnancy; severe pre-eclampsia with blood pressure at 160 − 120/100 − 80 mm Hg; heart rate 60–120 beats/min; gestational age at 35–39 week. We excluded women with BMI > 40 kg/m^2^ and with opioid allergy. Any contraindication to spinal or epidural anesthesia, including local infection or intracranial hypertension, coagulation abnormality, platelet count < 75 × 10^9^/L, local or generalized sepsis, chronic hypertension, cord prolapsed, twin pregnancy, active labor, or a non‑reassuring fetal heart rate.120 patients who met the inclusion and exclusion criteria were randomized into two groups at the end of surgery to receive intrathecal morphine combined with TAP block(TAP group) or intrathecal morphine alone with sham block(Sham group) using a random number table. Random allotment was done using a randomisation list from an internet software package (www.sealedenvelope.com) generated by an independent statistician. Patients were allocated sequentially to their groups as per numbered opaque envelopes.

### Spinal procedure

None of the patients had preoperative medication. All patients were given eight-hours routine and water fasting before surgery. On arrival in the operating room, intravenous access was opened, and cardiac monitoring and oxygen inhalation of 2–3 L/min were started prior to introduction to anesthesia. Spinal anesthesia was initiated in the left recumbent position at the L3 to L4 interspace.

After confirmation of clear cerebrospinal fluid flow, 15 mg of 0.5% ropivacaine (AstraZeneca Pharmaceutical Co., Ltd., China: NAKS) was injected with 0.1 mg of morphine (Northeast Pharmaceutical Group Co., Ltd., China; Lot No.: 190306-2). After the injection, the epidural tube was placed in reserve. Five minutes later, 3 ml of 2% lidocaine was added to adjust the anesthesia level reach to T6 if the level of anesthesia did not reach T6 on the premise of no signs of lumbar anesthesia and inadvertent vascularization were observed. Supplemental oxygen was delivered through a facemask at 3.0 L/min. When systolic blood pressure is 30% lower than basal systolic blood pressure, 0.5 mg of metaraminol was administered intravenously.atropine 0.5 mg was administered for HR < 50 beats/min. 5 mg of dexamethasone (Zhengzhou Zhuofeng Pharmaceutical Co., Ltd., China; Lot No.: H41020055) and 5 mg of toltestrone (China; Lot No. H20080750) was administered intravenously to prevent postoperative vomiting after delivery of baby.

### Postoperative analgesia management

All patients were connected to an intravenous patient-controlled analgesia (PCA, product model: ZZB-150) infusion device to receive opioid for postoperative analgesia. The PCA device was configured by a uniform formula with 100ug of sufentanil (Yichang Renfu Pharmaceutical, China; lot no.: H20054171), 10 mg of dizocine (Yangtze River Pharmaceutical Group Co., Ltd., China; Lot No.H20080329), 5 mg of toltestrone and 100ml of saline) set to a deliver a 0.6ml/10kg bolus with a lock-out time of 10 min. The PCA device was pressed when the maternal resting pain VAS score was ≥ 4.

### Tap block procedure

Patients were given 20 ml of 0.35% ropivacaine per side in the TAP group and the same volume of 0.9% saline in the Sham group under ultrasound guided at the end of surgery in the anesthesia recovery room according to the pre-assigned groups.

### Outcomes

Postoperatively, the investigator collected patient pain level at rest and with movement measured on a visual analog scale (VAS) pain score at 4,8,12,24 h after TAP block. The VAS pain score was from 0 to 10( 0 represent no pain, 0 to 3 represent mild pain and tolerable; 4 to 6 represent moderate pain and tolerable; and 7 to 10 represent severe pain and unbearable pain). Times of analgesic demand on PCA within 12 h after surgery represents opioid consumption and the data of were downloaded from the PCA device.

Other outcome included occurrence of side effects within 24 h after anesthesia(including chills, nausea, skin pruritus, and respiratory depression ), and Apgar score at 1 and 5 min of newborns. Respiratory depression was defined as respiratory rate less than 10 or oxygen saturation less than 90%[13]. Maternal satisfaction was asked to rate on a four point scale with the pain control (1 score represents dissatisfied, 2 score represents general satisfied, 3 score represents fairly satisfied and 4 score represents highly satisfied).

### Statistical analysis

Sample size was calculated using PASS version 15.0.5. A minimal sample size of 47 patients in each group was needed for a power level of 0.80, alpha level of 0.05 (two tailed), and according to the previous pilot study, the satisfaction of patients in group TAP was 95% and that in group Sham was 75%. To overcome the loss to follow up, the calculated sample size was increased by 20% to reach 118 participants in each group.

Statistical analysis was performed using IBM SPSS 22.0 software. The Shapiro-Wilk test was used to test the normality of distribution. Mean ± SDs was used to describe normally distribution data and median and interquartile range for non-normally distribution data. Differences between TAP and Sham groups were assessed by independent t-tests or Mann-Whitney U-test. Categorical variables were described as number(percentage) and compared using Chi-square tests. The VAS pain scores between groups were conducted using Prism8 (GraphPad, San Deigo,CA, USA). *P* < 0.05 was considered a statistically significant difference.

## Results

**Fig. 1:**
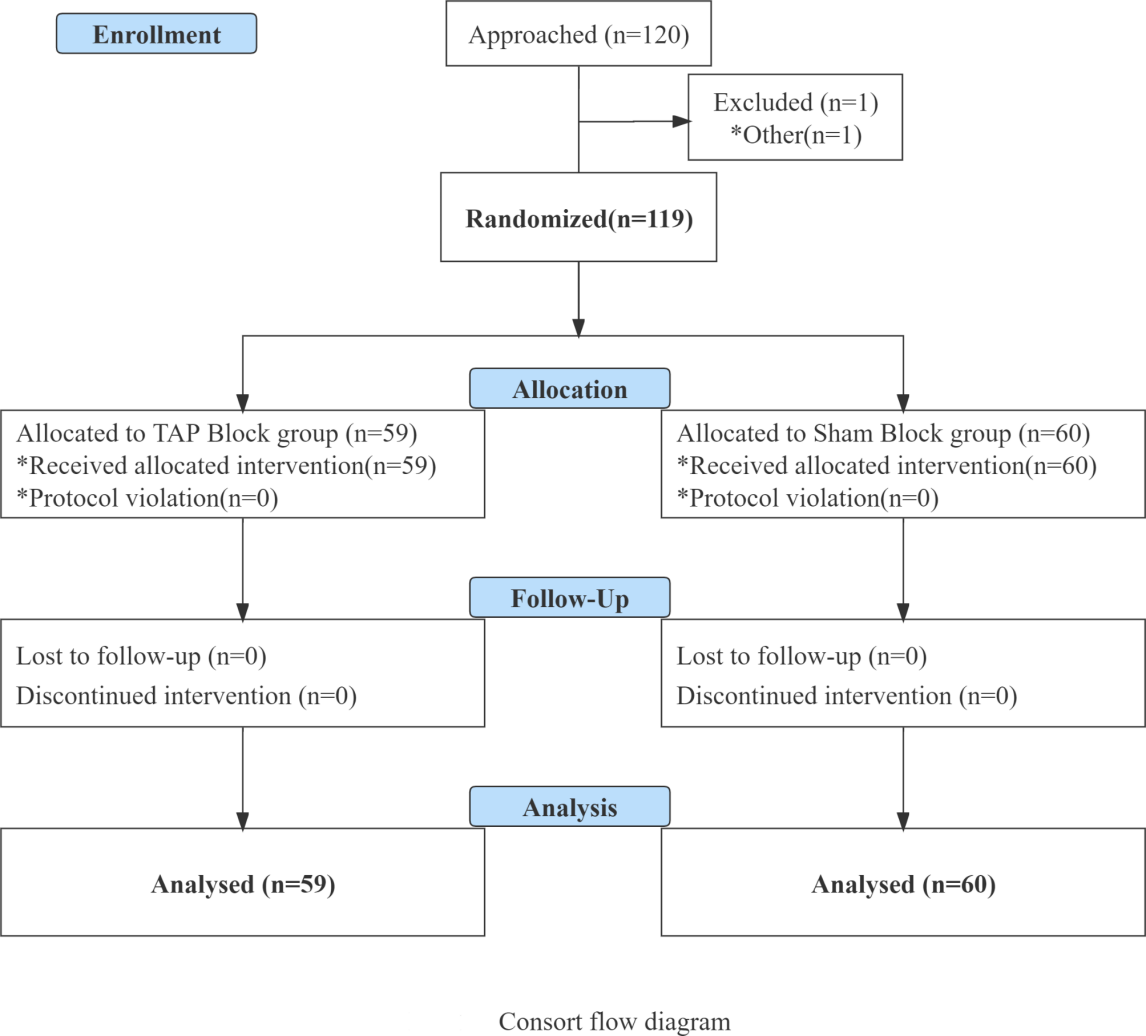
Consort flow diagram

Sixty patients were enrolled for each group(Fig. 1). One patient from group TAP was excluded because of poor anesthesia requiring adjunctive drug administration. Finally, a total of 119 patients including 60 patients in the Sham group and 59 patients in the TAP group received the intervention and the related data were analyzed. Patient’s baseline characteristics are reported in Table 1. There were no significant differences found in the maternal age, height, weight, and body mass index (BMI) between groups(*P*>0.05). VAS pain scores at four-time intervals are presented in Table 2. The median (interquartile range )of VAS pain score at rest in the TAP group and Sham group, respectively were: at 4 h, 1(0,1) vs. 1[1, 2], *P < 0.001*; at 8 h, 1[1] vs. 1.5[1, 2],*P < 0.001*; at 12 h,1[1, 2] vs. 2[1, 2],*P = 0.001*; and at 24 h, 2[1, 2] vs. 2(1,2.75),*P = 0.498*. Similarly, the median (interquartile range )of VAS pain scores with movement in the TAP group and Sham group, respectively were: at 4 h, 2[2, 3] vs. 3(2,3.75),*P = 0.062*; at 8 h, 2[2, 3] vs. 3[2, 4], *P = 0.060*; at 12 h, 3[2, 4] vs. 3[2, 4],*P = 0.364*; and at 24 h, 5[3, 5] vs. 4(2.25,5) *P = 0.324*. VAS pain scores at rest at 4,8,12 h were significantly different between groups but no significant difference at 24 h at rest and at all time points with movement. The maternal analgesia satisfaction is reported in Table 3. There were 36 (61.0%)patients in the TAP group who felt highly satisfied with pain control and 6(10%) patients in the Sham group, *P* < 0.05. The total proportion of maternal satisfaction with pain control in TAP group (n = 53,89.8%)were significantly higher in Sham group(45,75.0%),*P* < 0.05.

**Table 1:** Basic characteristics of participant

	TAP group(n = 59)	Sham group(n = 60)
Age(year)	30.49 ± 3.49	29.62 ± 3.27
Body Weight(kg)	67.37 ± 5.04	67.95 ± 5.38
Body Height(cm)	161.58 ± 4.34	161.49 ± 3.86
BMI	25.83 ± 2.02	26.07 ± 2.05

TAP:Transversus abdominis plane block in conjunction with intrathecal morphine use

Sham: intrathecal morphine alone group

BMI:body mass index

Values are present as mean ± SD

**Table 2:** Comparison Between TAP group and Sham group According to Visual Analog Scale (VAS) at Rest and with Movment

VAS		TAP group (n = 59)		Sham group (n = 60)		*P value*
		Median	IQR	Median	IQR	
4 h	At rest	1	(0–1)	1	(1–2)	*<0.001*
postoperatively	With movement	2	(2–3)	3	(2-3.75)	*0.062*
8 h	At rest	1	(1–1)	1.5	(1–2)	*<0.001*
postoperatively	With movement	2	(2–3)	3	(2–4)	*0.060*
12 h	At rest	1	(1–2)	2	(1–2)	*0.001*
postoperatively	With movement	3	(2–4)	3	(2–4)	*0.364*
24 h	At rest	2	(1–2)	2	(1-2.75)	*0.498*
postoperatively	With movement	5	(3–5)	4	(2.25-5)	*0.324*

Abbreviations: VAS, visual analog scale;

TAP:Transversus abdominis plane block in conjunction with intrathecal morphine use

Sham: intrathecal morphine alone group

**Table 3:** Times of analgesic demand on PCA, side effects, maternal satisfaction and Apgar score at 1min and 5min in groups

		TAP group (n = 59)	Sham group (n = 60)
Times of analgesic demand on PCA within 12 h after surgery		1.7 ± 1.1	2.0 ± 1.7
Maternal side effects	chills	6(10.2)	8(13.3)
	nausea	6(10.2)	5(8.3)
	skin pruritus	19(31.1)	18(30.0)
	respiratory depression	0(0.0)	0(0.0)
Maternal satisfaction*	Dissatisfied	1(1.7)	1(1.7)
	General satisfied	5(8.5)	14(23.3)
	Fairly satisfied	17(28.8)	39(65.0)
	Highly satisfied	36(61.0)	6(10.0)
Apgar score	1 min	9.4 ± 0.8	9.3 ± 0.8
	5 min	9.9 ± 0.3	9.9 ± 0.4

Data are shown as Mean SD for continuous variables and N(%) for categorical variables

P value were calculated with the variance(ANOVA) for continuous variables and with Chi-square test for categorical variables

* P value < 0.05

The mean(SD) times of analgesic demand on PCA within 12 h after anesthesia in the TAP group was 1.7(1.1), failed less than the Sham group of 2.0(1.7), *P* > 0.05. That is, the difference in opioid consumption at 12 h was not significant. There were no significant difference in the incidence of side effects, for chills was 6(10.2%) vs. 8(13.3%) ,*P = 0.287*; for nausea was 6(10.2%) vs. 5(8.3%),*P = 0.120*; and for skin pruritus was 19(31.1%) vs. 18(30.0%),*P = 0.067* in the TAP group and Sham group, respectively. The itching mainly focused on the face and was often self-limiting and did not require medical treatment. None of the patients in the study population had respiratory depression. The Apgar scores of the two groups at 1 and 5 min were not significantly different (*P* > 0.05).

## Discussion

Cesarean delivery is often performed in high-risked pregnancies to reduce the morbidity and mortality rate of high-risk mothers and newborns. However, the procedure is invasive and is often associated with severe postoperative incisional pain, which can have an impact on maternal endocrinology and postoperative recovery. Intrathecal morphine provides prolonged postoperative analgesia and reduces the amount of postoperative intravenous analgesics, and also significantly reduces the incidence of chronic pain and postpartum depression after cesarean delivery. Intrathecal morphine has a capping effect, and its safe dose is 50-200ug. Side effects are dose-related, specifically respiratory depression, hypotension, nausea and vomiting, skin pruritus, and so on[12, 13]. Therefore, to reduce the side- effects of morphine, other analgesic methods such as transverse abdominal fascial plane block, epidural analgesia, and intravenous analgesia are often required. Therefore, it is essential for women with severe pre-eclampsia to provide perfect postoperative analgesia after a cesarean section.

This prospective randomized trial was designed to compare the use of TAP block in conjunction with intrathecal morphine versus intrathecal morphine alone in women with severe pre-eclampsia. The results showed that combined use of intrathecal morphine and TAP block could reduce VAS pain scores at 4,8 and 12 h after surgery at rest. This finding was contradicted by those of Costelloet al[14], who found the TAP block used as part of a multimodal regimen inclusive of intrathecal morphine does not improve analgesia efficacy after cesarean delivery but is similar with the research of Terry T[15]. Superior analgesia management will benefit to lower morbidity and mortality as severe pain can lead to myocardial ischemia and is detrimental to keeping blood pressure stable. We found no differences in the VAS pain score at 24 h after surgery at rest and four-time points with movement. We infer that with the prolongation of postoperative time, the sensitivity of subjective pain decreased. As for no difference in VAS pain score during movement, this may be because both two groups receive self-administered of opioid from PCA device when the pain sensation increases during movement.

The incidence of side effects was not statistically different in both groups which may be due to the not enough sample size or related to our study protocol. Our saline-based sham block may create a placebo effect for women, resulting in no difference in the incidence of side effects.

Most patients rated their satisfaction with pain control as good and the total incidence of satisfaction in the TAP group was higher than that in the Sham group, which suggested an additional analgesic effect may have been gleaned from the TAP block. The use of TAP block did not result in a reduction in opioid consumption from the PCA device in the first 12 h and this finding was different from the previous research[16–18]. In their study on patients with or without TAP after cesarean section, the morphine consumption is lower when the TAP block is used. It’s worth noting that in their study, opioid consumption from PCA device was counted up to 24 h postoperatively, while our study only up to 12 h which may have contributed to the lack of difference in opioid consumption.

The TAP block is a local anesthetic injected into the neurofascial layer between the internal oblique and transversus abdominis muscles to block the anterior abdominal wall T6-L1 segmental nerve, which provides good analgesia for abdominal wall incision, reduces pain scores and opioid dosage and is beneficial for rapid postoperative recovery, and is an important component of multimodal analgesia[19].

The benefit of TAP block for postoperative analgesia is evident in patients undergoing cesarean section without intrathecal morphine. It reduces opioid consumption in PCA devices and is safe with few side effects.In patients who had taken intrathecal morphine, Kendall et al[20]. concluded that TAP block did not increase the efficacy of their post-cesarean analgesia. Salama et al[21]. compared the effect of lumbar square muscle block with intrathecal morphine on analgesia after cesarean section and showed that lumbar square muscle block reduced VAS pain scores at 12 and 24 h postoperatively, reduced opioid consumption from PCA device at 12 h postoperatively and prolonged the time to first morphine administration. In this study TAP, although it did not significantly reduce the number of pressures for analgesia at 24 h postoperatively, significantly reduced the VAS pain scores for rest and movement at 12 h postoperatively and increased the probability of very satisfactory maternal outcomes. It illustrates the synergistic effect of TAP compounded with intrathecal morphine and postoperative PCA analgesia in severe pre-eclampsia cesarean section.

There are some limitations to our study. Firstly, we take the VAS pain score of the patients as the primary outcome which mainly is due to the different tolerance and sensitivity to the pain of each patient. Secondly,There is potential to use a higher concentration with a volume increased to the maximum safe dose. Further studies using different approaches for TAP or increasing concentrations of local anaesthetics with adjuncts to prolong analgesic duration are warranted.Thirdly,this study is a single-center study with a small sample size, and there are region and race limitations. Although these limitations, our study perform an actual TAP block with saline rather than a simulated sham injection can increase the internal validity of the investigation. In the future, we will expand the sample size and include multi-center to explore more multimodal analgesia to seek the best postoperative analgesia.

## Conclusions

In conclusion, The TAP block performed in conjunction with intrathecal morphine may not reduce opioid consumption, but it could reduce VAS pain scores at rest in the first 12 h after cesarean section in women with severe pre-eclampsia, and improve maternal satisfaction, which is worthy of clinical promotion.

## Data Availability

The datasets used and/or analysed during the current study are available from the corresponding author on reasonable request.

## Abbreviations

TAP: Transversus abdominis plane
VAS: Visual analog scale
PCA: Patient-controlled analgesia
TAP group: Transversus abdominis plane block in conjunction with intrathecal morphine use group
Sham group: Intrathecal morphine alone group
